# Expansion Light Sheet Microscopy Resolves Subcellular Structures in Large Portions of the Songbird Brain

**DOI:** 10.3389/fnana.2019.00002

**Published:** 2019-01-31

**Authors:** Daniel Normen Düring, Mariana Diales Rocha, Falk Dittrich, Manfred Gahr, Richard Hans Robert Hahnloser

**Affiliations:** ^1^Institute of Neuroinformatics, University of Zürich/ETH Zürich, Zurich, Switzerland; ^2^Neuroscience Center Zurich (ZNZ), Zurich, Switzerland; ^3^Department of Behavioral Neurobiology, Max Planck Institute for Ornithology, Seewiesen, Germany

**Keywords:** expansion microscopy, tissue clearing, songbird, spine morphology, light sheet microscopy, large volume imaging, super resolution microscopy

## Abstract

Expansion microscopy and light sheet imaging (ExLSM) provide a viable alternative to existing tissue clearing and large volume imaging approaches. The analysis of intact volumes of brain tissue presents a distinct challenge in neuroscience. Recent advances in tissue clearing and light sheet microscopy have re-addressed this challenge and blossomed into a plethora of protocols with diverse advantages and disadvantages. While refractive index matching achieves near perfect transparency and allows for imaging at large depths, the resolution of cleared brains is usually limited to the micrometer range. Moreover, the often long and harsh tissue clearing protocols hinder preservation of native fluorescence and antigenicity. Here we image large expanded brain volumes of zebra finch brain tissue in commercially available light sheet microscopes. Our expansion light sheet microscopy (ExLSM) approach presents a viable alternative to many clearing and imaging methods because it improves on tissue processing times, fluorophore compatibility, and image resolution.

## Introduction

Since the advent of modern neuroscience, diverse imaging techniques have allowed us to see the brain's microstructure and have fueled our endeavor to understand its function. With the inherent desire to see even the smallest relevant cellular building blocks, the preservation of detailed topographic information within entire neural circuits becomes challenging. Recent advances demonstrate the feasibility of large volume high-resolution imaging with light (Kleinfeld et al., 2011) or electrons (Kasthuri et al., 2015).

The desire to image entire intact volumes of tissue, a daunting task (Marblestone et al., 2013), is mirrored in the recent explosion of tissue clearing protocols (Richardson and Lichtmann, 2015, 2017).

Clearing protocols strive for deeper light penetration by homogenizing light refraction throughout the sample. These protocols make use of diverse chemical approaches from simple immersion to organic solvent-based delipidation (Susaki and Ueda, 2016). Nearly all clearing protocols sufficiently increase transparency for imaging multiple millimeters deep into tissues. However, targeted fluorescence imaging remains challenging because genetically expressed fluorescent proteins tend to lose their fluorescence and their antigenicity during tissue clearing. Recent progress in clearing protocols have led to better preservation of fluorescence (CUBIC; Susaki et al., 2015) or to improved post-immunostaining (CLARITY; Chung and Deisseroth, 2013; Tomer et al., 2014 and iDISCO; Renier et al., 2014), but current protocols are either time consuming and/or resource intense, technically challenging, and/or impractical. For excellent overviews, see Richardson and Lichtmann (2015, 2017) and Silvestri et al. (2016), who conclude that no single protocol is superior to all others.

One technique that could address many of the aforementioned shortcomings is expansion microscopy (ExM). In ExM, samples are first incubated with DMSO to introduce acryloyl groups. Then, the proteins (including fluorophores) are cross-linked to a polymer grid. After a protein digestion step, the sample is physically expanded through hyper-hydration (Chen et al., 2016; Chozinski et al., 2016; Tillberg et al., 2016). The increase in resolution resulting from expansion has pushed the limits of confocal microscopy and enabled the investigation of subcellular structures that previously have been difficult to resolve by light microscopy, such as the synaptonemal complex in Drosophila (Cahoon et al., 2017). Further, expansion microscopy has proven to be useful in clinical settings by enhancing the resolving power of microscopes (Bucur et al., 2016; Zhao et al., 2017). Although the clearing potential of the expansion protocol and its use for larger specimens have been emphasized (Chen et al., 2015; Richardson and Lichtmann, 2015; Karagiannis and Boyden, 2018), to date ExM has only been applied to tissue samples <200 μm thick from mice and humans.

When large volumes of tissue are to be imaged, preferably with multiple wavelengths, image acquisition times, system stability, and photo bleaching become increasingly problematic (Silvestri et al., 2016). One recent technological revival in fluorescence microscopy, the light sheet microscope, alleviates some of these challenges. By illuminating entire planes of tissue and capturing all image pixels at the same time, imaging is fast and fluorophores show little bleaching (Stelzer, 2015).

Here we combine expansion microscopy of large tissue volumes with light sheet microscopy, termed ExLSM. We demonstrate the feasibility of ExLSM in a small passerine, the zebra finch, and point out some advantages over other clearing methods.

Zebra finches are a well-established model for vocal learning with parallels to human speech acquisition (Bolhuis et al., 2010). Furthermore, the organization of the song system, responsible for song learning and production in songbirds, as an interconnected network of discrete brain nuclei makes it particularly well-suited for the analysis of cerebral sub-volumes (Vicario, 1991). Song system nuclei are known to adapt in response to song learning and practice (Huang et al., 2018), making the application of large tissue ExLSM particularly promising within this field, as discrete nuclei can be dissected and expanded to further analyze connectivity changes in detail. We focus on two nuclei of the song system, HVC (proper name), which drives vocal sequences (Hahnloser et al., 2002) and Area-X, which plays an essential role during vocal learning (Scharff and Nottebohm, 1991).

## Methods

### Tissue Preparation

Five adult male zebra finches have been intracranially injected into either HVC or Area-X with adeno-associated viruses (AAVs) that express GFP in the cytoplasm of neurons. After isoflurane overdose and transcardial perfusion with phosphate-buffered saline, hemispheres were either sectioned into 60–100 μm thick sections (*n* = 2 birds) using a microtome (Leica Microsystems, Germany), sectioned into 300–800 μm thick sections containing HVC using a vibratome (*n* = 2), or were handled to surgically remove entire HVCs (*n* = 1).

Animal handling was carried out in accordance with the European Communities Council Directive 2010/63 EU and legislation of the state of Upper Bavaria.

The government of Upper Bavaria, “Sachgebiet 54—Verbraucherschutz, Veterinärwesen, 80538 München” with the record number 55.2-1-54-2532-150-2016 approved animal experiments.

### Expansion and Clearing

We performed the protein retention expansion protocol (proExM) available at expansionmicroscopy.org with minor modifications to accommodate large tissues. In brief, the activation step needs to be adjusted according to tissue volume. Custom gelation chambers can be constructed by sectioning a 1 ml pipette tip according to size and placing them on a parafilm wrapped object slide for easy handling. Immediately after digestion any movement of the sample should be minimized or avoided. Expansion should be performed in the final imaging container if possible. Large gels can be secured with 3% low melt agarose around the corners of the gel. Small gels or hanging gels can be embedded in 1% low melt agarose. For further details and handling recommendations see Supplementary Materials.

### Imaging

Images were acquired in commercially available light sheet microscopes, either the Ultra Microscope II (LaVision Biotec GmbH, Germany) or the Z.1 (Carl Zeiss AG, Germany). Image analysis was performed using Imaris (Bitplane, Great Britain). With exception of the image in **Figure 2F**, which has been deconvolved using Huygens Pro (Scientific Volume Imaging B.V., Netherlands), none of the data has undergone any image preprocessing prior to analysis as is common practice in light based imaging.

## Results

**Figure 1:**
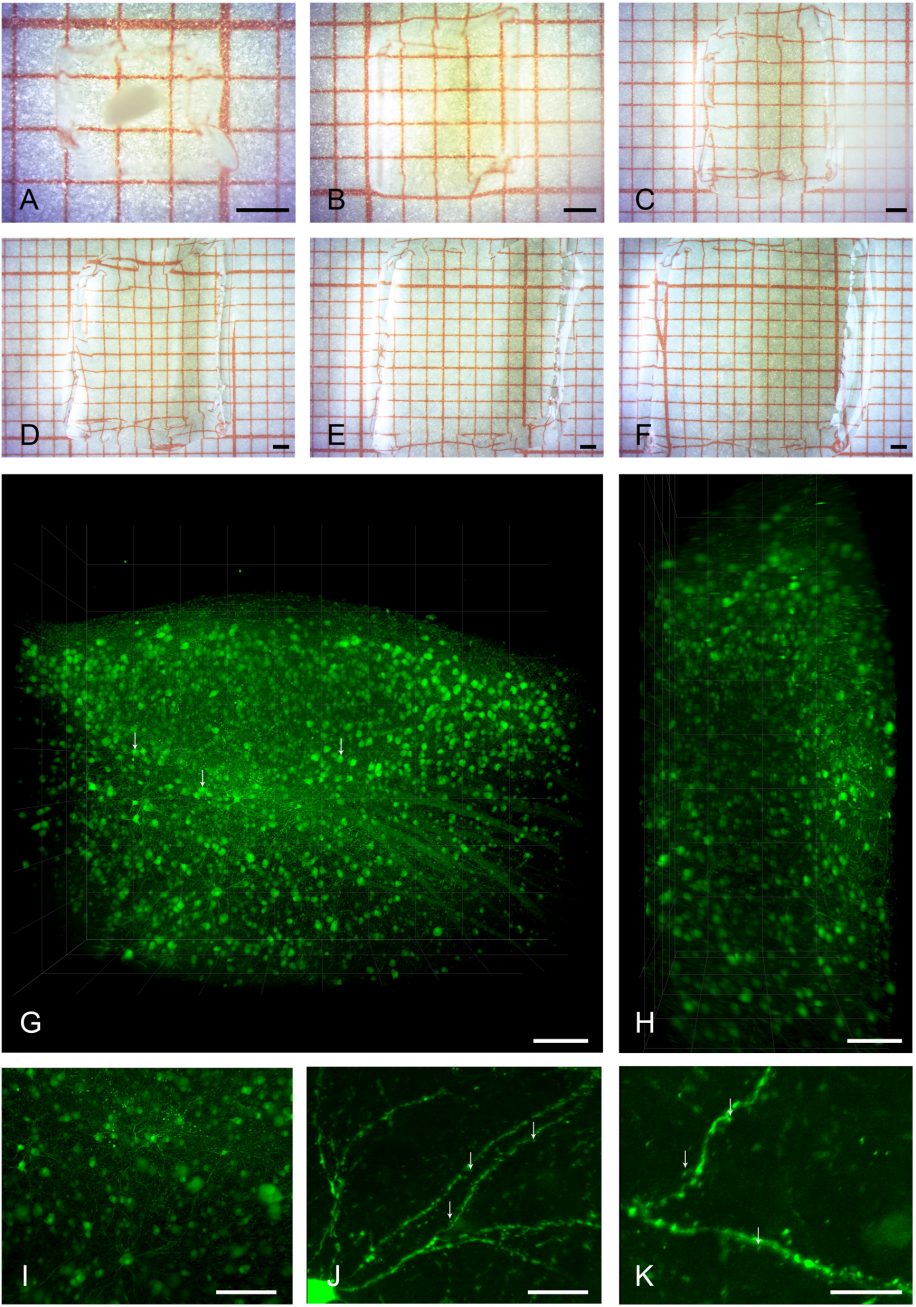
Clearing, expansion, and imaging of large intact volumes of brain tissue. Top two rows show the typical clearing and swelling progress of the expansion microscopy protocol. A large piece of brain tissue containing HVC is embedded in a polymer gel **(A)**, and is neither expanded nor transparent at this point. Immediately after taking the gel out of the protein digestion solution **(B)**, the tissue is already fully transparent and slightly expanded in volume by a factor of two. At this stage, the tissue can be kept in PBS for either imaging, further processing, or storage. Subsequent exchanges of deionized water **(C–F)** lead to a final 4-fold expansion (64 times in volume). Bottom two rows show volume renderings of brain tissue imaged with the LaVision Ultra Microscope II. **(G)** Shows the entire volume of a gel containing a piece of tissue with pre-expansion dimensions of [2500 × 1800 × 800] μm^3^, including a large part of HVC. Somata of neurons and even some sub-cellular structures such as nuclei (arrows) are readily visible at a low-magnification setting of 2.5x. Even though the light sheet leads to a slight reduction in axial resolution as seen in the xz-projection view in **(H)**, individual neurons can still be discriminated. A digital zoom into the dataset **(I)** reveals some axonal and dendritic processes. The entire dataset was imaged as a single TIFF stack with a z-step size of 3 μm. Imaging at slightly higher magnification of 6.4x allows for the identification of spiny dendrites (arrows, **J,K**). Scale bars **(A–F)** 1 mm, **(G,H)** 250 μm, **(I)** 150 μm, **(J)** 75 μm, **(K)** 50 μm. Scale bars in **(G–K)** correspond to pre-expansion dimensions.

We successfully cleared and expanded adult zebra finch brain tissue of diverse volumes ranging from 60 μm thick sagittal brain sections to [2500 × 2000 × 1200] μm^3^ tissue volumes encapsulating an entire HVC (Figures 1A–F).

Samples were subsequently imaged in two different light sheet microscopes, first a low-magnification system for overview and integral volume analysis, and, second, a system with a smaller working range but higher magnification.

### Ultramicroscope II for Large Samples

Figures 1G–I and Supplementary Movie S1 show a volume rendering of a tissue block with pre-expansion dimensions of [2500 × 2000 × 800] μm^3^. Somata of HVC neurons are easily identifiable and axonal structures can be recognized even at low magnification (Supplementary Figure S2). Under increased magnification, fine structures such as spines can be discerned (Figures 1J,K).

### Z.1 for High Resolution Imaging

**Figure 2:**
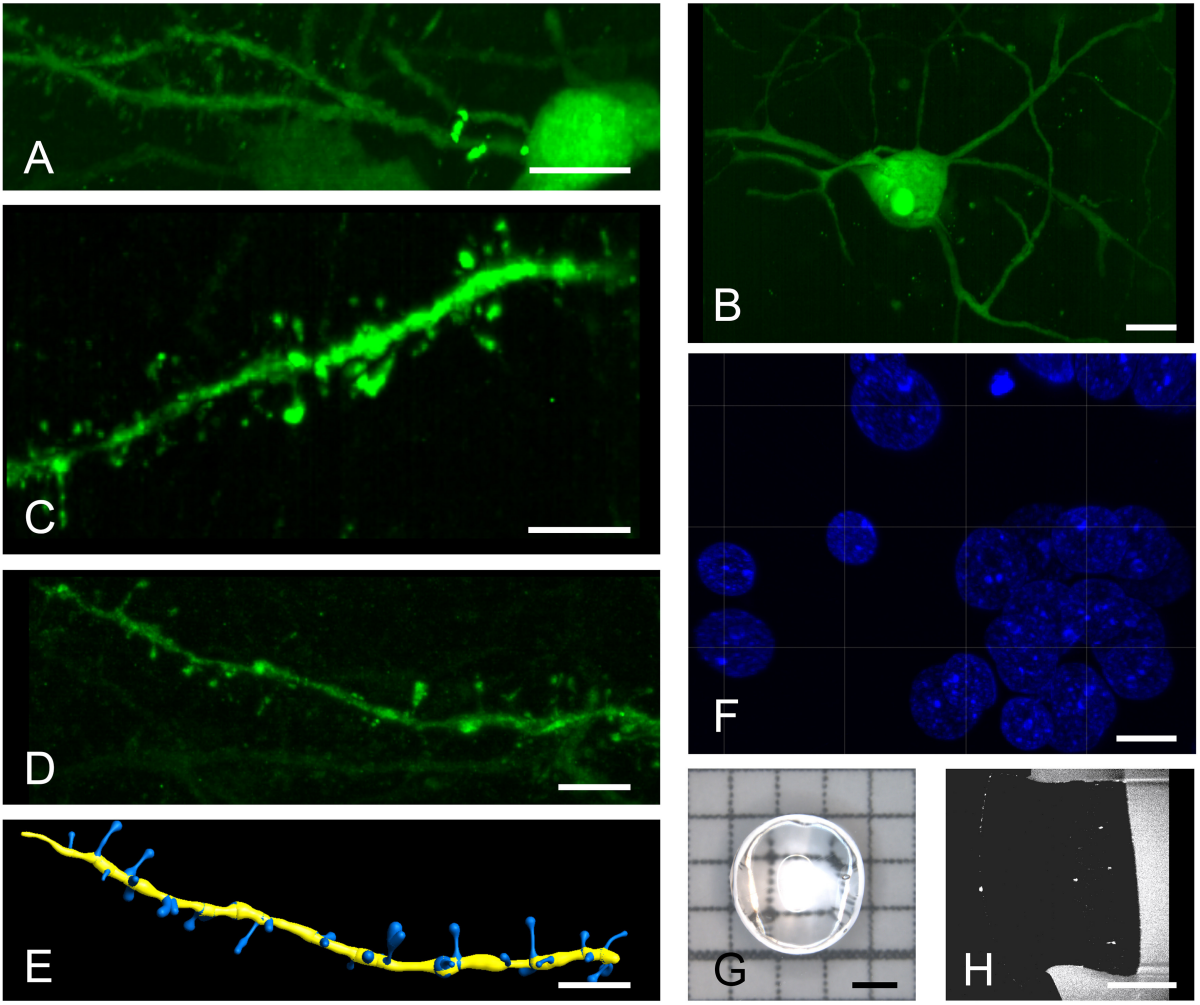
High resolution imaging of spiny and aspiny neurons. Top row shows a spiny **(A)** and an aspiny **(B)** neuron in Area X. Higher magnifications reveal distinct spine morphologies **(C,D)** that can be automatically quantified using the IMARIS filament tracer **(E)**. A brief post-expansion DAPI staining **(F)** resolves cellular nuclei with labeled chromatin structures. We imaged 3 mm punch-outs **(G,H)** of 4-fold expanded, 60–100 μm thick (pre-expansion) sagittal brain sections containing almost the entire Area X. All images are produced with a 20x, 1.0 NA water dipping objective. Scale bars **(A,B)** 10 μm, **(C–F)** 5 μm, **(G,H)** 1 mm. Scale bars in **(A–F)** correspond to pre-expansion dimensions.

For analysis requiring higher resolution, we turned to a system that is equipped with regular microscope objectives. Images acquired with a 20x objective distinctly show surface structures of somata and dendrites in both spiny (Figure 2A) and aspiny (Figure 2B) neurons, which can be easily discriminated. Digitally zoomed-in maximum intensity projections of diverse dendrite fragments clearly reveal spines (Figures 2C,D) of distinct morphologies (Figure 2E). General morphology and a spine density of 0.70 ± 0.09 (*n* = 6) per μm dendrite length match previous descriptions of HVC-X projecting neurons using EM (Kornfeld et al., 2017).

Post-expansion staining with DAPI shows exceptional details of the nucleus (Figure 2F) with image quality exceeding that of cell cultures imaged with gSTED (Okada and Nakagawa, 2015). We typically imaged 3 mm punch-outs of expanded sagittal brain sections of Area X (Figures 2G,H).

## Discussion

We demonstrated the clearing capacities of our expansion protocol and showed that ExLSM allows for imaging of large volumes of brain tissue at subcellular resolution. In the following, we compare our approach to other clearing approaches. For a more in-depth review of cleared-sample imaging, we refer to Richardson and Lichtmann (2015, 2017) and to Silvestri et al. (2016); for a focus on clearing of songbird tissue, we refer to our other publication in the same research topic (Rocha et al., 2019).

### Transparency and Large Volume Imaging

A general limitation of light-based imaging techniques such as confocal microscopy is the increase in light scattering with tissue depth when imaging non-cleared specimens. The increase in background noise makes imaging at depths larger than 50 μm impractical. Homogenization of refractory indices (RIs) across sample, embedding medium, and imaging medium is one common goal to all clearing methods (Silvestri et al., 2016). With ExLSM, commonly no tissue outlines are visible and gels themselves are completely transparent in water, which hints toward a near perfect match of RIs throughout the optical path. High tissue clarity is suggested by the extremely low levels of background light. Another advantage of water as imaging medium over solutions with different RIs is compatibility with imaging objectives, because no additional corrections in the imaging path are necessary.

Although we demonstrated the unobstructed imaging of cleared gel blocks of roughly one cubic centimeter, we emphasize that our gels contained biologically relevant data within only 2 mm^3^ pre-expansion. Whether the expansion and excellent clearing of much larger volumes is feasible remains to be tested.

Another factor to consider when imaging cleared tissue in a light sheet microscope is the color of the fluorophore. Because light of longer wavelength is less affected by scattering, the use of red fluorophores is generally advised, such as DRAQ5 as a nuclear marker instead of DAPI (Marx, 2016). Here, we demonstrated the compatibility of ExLSM with short-wavelength fluorophores.

### Retention of Native Fluorescence and Antigenicity

In general, organic solvent-based clearing methods do not preserve native fluorescence of proteins well, with the exception of uDISCO (Pan et al., 2016). GFP fluorescence is usually well preserved, but we want to draw attention to the fact that the common model for assessing fluorescence preservation, the Thy-1 mouse line, has an exceptionally strong GFP signal (Dodt et al., 2007; Silvestri et al., 2016). Many protocols are to some degree compatible with antibody staining, but antibody penetration based on passive diffusion can take weeks (Richardson and Lichtmann, 2015). Additional usage of electrophoresis such as in CLARITY (Chung et al., 2013) or in PARS (Yang et al., 2014), or the use of pressure as in ACT-PRESTO (Lee et al., 2016) can accelerate antibody penetration, but these enhancements can be difficult to set up or expensive to use when turning to a commercial solution such as X-CLARITY™ (Logos Biosystems, South Korea). Expansion microscopy, on the other hand, allows for post-expansion staining and is readily compatible with a large number of genetically expressed fluorescent proteins and commercially available antibodies (Chozinski et al., 2016; Ku et al., 2016; Tillberg et al., 2016).

To stain in expanded tissues is a potentially very powerful advantage. By stretching and unfolding, proteins' epitopes may become more accessible (Alon et al., 2018), which can accelerate antibody penetration and increase labeling strength.

### Image Resolution

One important consideration for imaging is resolution. In light sheet microscopy, axial resolution is directly proportional to the physical thickness of the light sheet and lateral resolution depends on the imaging objective. When pushing the physical limits, bessel beam-based systems and lattice light sheets present the current pinnacle of high axial resolution with voxel sizes of 150 × 150 × 280 nm (Chen et al., 2014), but at the cost of smaller working distances associated with the high NA illumination and imaging objectives used.

Desired resolution is of course dependent on the scientific question at hand. Hence, the 4–5 μm axial resolution that is achievable with conventional illumination lenses might be sufficient and even favorable for the sake of working distance. For example, considering the Nyquist criterion, isotropic resolution of 5 μm is enough to resolve cell bodies with a diameter larger than 10 μm, which is generally the case when looking at neurons. Although some clearing approaches can visualize dendritic spines in 2D with higher NA imaging objectives (Dodt et al., 2007), quantification of structure in 3D remains challenging. Even the recent addition to the CUBIC protocol, CUBIC-X (Murakami et al., 2018), that exploits the intermediate expansion of the protocol's reagent-1 to achieve a final 2-fold increase in volume, does not seem to allow for the distinct differentiation of spine morphologies (compare Figure 2H in Murakami et al., 2018).

We demonstrate that spine morphology can be quantified with ExLSM in a Zeiss Z.1 light sheet microscope with results comparable to conventional slice-based confocal microscopy, with the advantage of increased imaging speed. Whereas, imaging a volume of [100 × 100 × 10] μm^3^ takes minutes in a confocal microscope, the same volume of [400 × 400 × 40] μm^3^ is imaged in seconds with ExLSM at a comparable resolution (Supplementary Figure S3). Considering a working distance of 2.4 mm for the 20x, 1.0 NA water-dipping objective used here, even quantification of spine morphologies in about 2 mm post-expansion sections is possible (corresponding to a native thickness of 500 μm).

## Conclusion

We have demonstrated the viability of expansion microscopy in the context of cleared large volume tissue imaging. The extremely short, easy, and inexpensive pre-processing in combination with great preservation of endogenous fluorescence, the possibility for post-staining, and its scalability make ExLSM a candidate for volumetric fluorescence imaging. We hope that this study encourages other labs to adapt our ExLSM approach to make imaging of large cleared and expanded volumes of brain tissue common practice in neural circuit analyses.

## Data Availability Statement

Datasets are Available online at: https://figshare.com/s/11103cff90436aa22630.

## Author Contributions

All authors conceived of the study. MG and RH provided instruments, materials, and reagents. DD, MR, and FD contributed to experiments, imaging, and interpretation of results. DD generated all figures. DD and MR wrote the first draft of the manuscript. All authors contributed to manuscript revision and read and approved the submitted version.

### Conflict of Interest Statement

The authors declare that the research was conducted in the absence of any commercial or financial relationships that could be construed as a potential conflict of interest.

## References

[B1] Alon S. Huynh G. H. Boyden E. S. (2018). Expansion microscopy: enabling single cell analysis in intact biological systems. FEBS J. 10.1111/febs.14597. [Epub ahead of print]. 29938896 PMC6309752

[B2] Bolhuis J. J. Okanoya K. Scharff C. (2010). Twitter evolution: converging mechanisms in birdsong and human speech. Nat. Rev. Neurosci. 11, 747–759. 10.1038/nrn2931 20959859

[B3] Bucur O. Zhao Y. Boyden E. Beck A. H. (2016). Physical expansion of tissue microarrays for high-resolution imaging of normal and cancer samples with conventional microscopy. Cancer Res. 76. 10.1158/1538-7445.AM2016-4229

[B4] Cahoon C. K. Yu Z. Wang Y. Guo F. Unruh J. R. Slaughter B. D.. (2017). Superresolution expansion microscopy reveals the three-dimensional organization of the Drosophila synaptonemal complex. Proc. Natl. Acad. Sci.U.S.A. 114, E6857–E6866. 10.1073/pnas.1705623114 28760978 PMC5565445

[B5] Chen B. C. Legant W. R. Wang K. Shao L. Milkie D. E. Davidson M. W.. (2014). Lattice light-sheet microscopy: imaging molecules to embryos at high spatiotemporal resolution. Science 346:1257998. 10.1126/science.1257998 25342811 PMC4336192

[B6] Chen F. Tillberg P. W. Boyden E. S. (2015). Expansion microscopy. Science 347, 543–548. 10.1126/science.1260088 25592419 PMC4312537

[B7] Chen F. Wassie A. T. Cote A. J. Sinha A. Alon S. Asano S.. (2016). Nanoscale imaging of RNA with expansion microscopy. Nat. Methods 13, 679–684. 10.1038/nmeth.3899 27376770 PMC4965288

[B8] Chozinski T. J. Halpern A. R. Okawa H. Kim H. J. Tremel G. J. Wong R. O.. (2016). Expansion microscopy with conventional antibodies and fluorescent proteins. Nat. Methods 13, 485–488. 10.1038/nmeth.3833 27064647 PMC4929147

[B9] Chung K. Deisseroth K. (2013). CLARITY for mapping the nervous system. Nat. Methods 10, 508–513. 10.1038/nmeth.2481 23722210

[B10] Chung K. Wallace J. Kim S. Y. Kalyanasundaram S. Andalman A. S. Davidson T. J.. (2013). Structural and molecular interrogation of intact biological systems. Nature 497:332. 10.1038/nature12107 23575631 PMC4092167

[B11] Dodt H. U. Leischner U. Schierloh A. Jährling N. Mauch C. P. Deininger K.. (2007). Ultramicroscopy: three-dimensional visualization of neuronal networks in the whole mouse brain. Nat. Methods 4:331. 10.1038/nmeth1036 17384643

[B12] Hahnloser R. H. Kozhevnikov A. A. Fee M. S. (2002). An ultra-sparse code underliesthe generation of neural sequences in a songbird. Nature 419, 65–70. 10.1038/nature00974 12214232

[B13] Huang Z. Khaled H. G. Kirschmann M. Gobes S. M. Hahnloser R. H. (2018). Excitatory and inhibitory synapse reorganization immediately after critical sensory experience in a vocal learner. Elife 7:e37571. 10.7554/eLife.37571 30355450 PMC6255392

[B14] Karagiannis E. D. Boyden E. S. (2018). Expansion microscopy: development and neuroscience applications. Curr. Opin. Neurobiol. 50, 56–63. 10.1016/j.conb.2017.12.012 29316506 PMC5984670

[B15] Kasthuri N. Hayworth K. J. Berger D. R. Schalek R. L. Conchello J. A. Knowles-Barley S.. (2015). Saturated reconstruction of a volume of neocortex. Cell 162, 648–661. 10.1016/j.cell.2015.06.054 26232230

[B16] Kleinfeld D. Bharioke A. Blinder P. Bock D. D. Briggman K. L. Chklovskii D. B.. (2011). Large-scale automated histology in the pursuit of connectomes. J. Neurosci. 31, 16125–16138. 10.1523/JNEUROSCI.4077-11.2011 22072665 PMC3758571

[B17] Kornfeld J. Benezra S. E. Narayanan R. T. Svara F. Egger R. Oberlaender M.. (2017). EM connectomics reveals axonal target variation in a sequence-generating network. Elife 6:e24364. 10.7554/eLife.24364 28346140 PMC5400503

[B18] Ku T. Swaney J. Park J. Y. Albanese A. Murray E. Cho J. H.. (2016). Multiplexed and scalable super-resolution imaging of three-dimensional protein localization in size-adjustable tissues. Nat. Biotechnol. 34, 973–981. 10.1038/nbt.3641 27454740 PMC5070610

[B19] Lee E. Choi J. Jo Y. Kim J. Y. Jang Y. J. Lee H. M. (2016). ACT-PRESTO: rapid and consistent tissue clearing and labeling method for 3-dimensional (3D) imaging. Sci. Rep. 6:18631 10.1038/srep18631 26750588 PMC4707495

[B20] Marblestone A. H. Daugharthy E. Kalhor R. Peikon I. Kebschull J. Shipman S. (2013). Conneconomics: the economics of large-scale neural connectomics. bioRxiv 001214. 10.1101/001214

[B21] Marx V. (2016). Optimizing probes to image cleared tissue. Nat. Methods 13, 205–209. 10.1038/nmeth.3774 26914203

[B22] Murakami T. C. Mano T. Saikawa S. Horiguchi S. A. Shigeta D. Baba K.. (2018). A three-dimensional single-cell-resolution whole-brain atlas using CUBIC-X expansion microscopy and tissue clearing. Nat. Neurosci. 21:625. 10.1038/s41593-018-0109-1 29507408

[B23] Okada Y. Nakagawa S. (2015). Super-resolution imaging of nuclear bodies by STED microscopy, in Nuclear Bodies and Noncoding RNAs eds Nakagawa S. Hirose T. (New York, NY: Humana Press), 21–35. 10.1007/978-1-4939-2253-6_2 25555573

[B24] Pan C. Cai R. Quacquarelli F. P. Ghasemigharagoz A. Lourbopoulos A. Matryba P.. (2016). Shrinkage-mediated imaging of entire organs and organisms using uDISCO. Nat. Methods 13, 859–867. 10.1038/nmeth.3964 27548807

[B25] Renier N. Wu Z. Simon D. J. Yang J. Ariel P. Tessier-Lavigne M. (2014). iDISCO: a simple, rapid method to immunolabel large tissue samples for volume imaging. Cell 159, 896–910. 10.1016/j.cell.2014.10.010 25417164

[B26] Richardson D. S. Lichtmann J. W. (2015). Clarifying tissue clearing. Cell 162, 246–257. 10.1016/j.cell.2015.06.067 26186186 PMC4537058

[B27] Richardson D. S. Lichtmann J. W. (2017). SnapShot: tissue clearing. Cell 171, 496–496. 10.1016/j.cell.2017.09.025 28985569

[B28] Rocha M. D. Düring D. N. Bethge P. Voigt F. F. Hildebrand S. Helmchen F. (2019). Tissue clearing and light sheet microscopy: imaging the unsectioned adult zebra finch brain at cellular resolution. 10.3389/fnana.2019.00013 PMC6382697 30837847

[B29] Scharff C. Nottebohm F. (1991). A comparative study of the behavioral deficits following lesions of various parts of the zebra finch song system: implications for vocal learning. J. Neurosci. 11, 2896–2913. 10.1523/JNEUROSCI.11-09-02896.1991 1880555 PMC6575264

[B30] Silvestri L. Costantini I. Sacconi L. Pavone F. S. (2016). Clearing of fixed tissue: a review from a microscopist's perspective. J. Biomed. Opt. 21:081205. 10.1117/1.JBO.21.8.081205. 27020691

[B31] Stelzer E. H. (2015). Light-sheet fluorescence microscopy for quantitative biology. Nat. Methods 12, 23–26. 10.1038/nmeth.3219 25549266

[B32] Susaki E. A. Tainaka K. Perrin D. Yukinaga H. Kuno A. Ueda H. R. (2015). Advanced CUBIC protocols for whole-brain and whole-body clearing and imaging. Nat. Protoc. 10, 1709–1727. 10.1038/nprot.2015.085 26448360

[B33] Susaki E. A. Ueda H. R. (2016). Whole-body and whole-organ clearing and imaging techniques with single-cell resolution: toward organism-level systems biology in mammals. Cell Chem. Biol. 23, 137–157. 10.1016/j.chembiol.2015.11.009 26933741

[B34] Tillberg P. W. Chen F. Piatkevich K. D. Zhao Y. Yu C. C. J. English B. P.. (2016). Protein-retention expansion microscopy of cells and tissues labeled using standard fluorescent proteins and antibodies. Nat. Biotechnol. 34, 987–992. 10.1038/nbt.3625 27376584 PMC5068827

[B35] Tomer R. Ye L. Hsueh B. Deisseroth K. (2014). Advanced CLARITY for rapid and high-resolution imaging of intact tissues. Nat. Protoc. 9, 1682–1697. 10.1038/nprot.2014.123 24945384 PMC4096681

[B36] Vicario D. S. (1991). Organization of the zebra finch song control system: functional organization of outputs from nucleus robustus archistriatalis. J. Comp. Neurol. 309, 486–494. 10.1002/cne.903090405 1655832

[B37] Yang B. Treweek J. B. Kulkarni R. P. Deverman B. E. Chen C. K. Lubeck E.. (2014). Single-cell phenotyping within transparent intact tissue through whole-body clearing. Cell 158, 945–958. 10.1016/j.cell.2014.07.017 25088144 PMC4153367

[B38] Zhao Y. Bucur O. Irshad H. Chen F. Weins A. Stancu A. L.. (2017). Nanoscale imaging of clinical specimens using pathology-optimized expansion microscopy. Nat. Biotechnol. 35, 757–764. 10.1038/nbt.3892 28714966 PMC5548617

