# Supplementary material for: Expansion Light Sheet Microscopy Resolves Subcellular Structures in Large Portions of the Songbird Brain
Source: Front Neuroanat. 2019 Jan 31;13:2. doi: 10.3389/fnana.2019.00002 (PMC6365838; doi:10.3389/fnana.2019.00002)
Supplement: Supplementary file 2 [file Data_Sheet_1.pdf]

Supplementary Material

Detailed expansion and clearing protocol

For expansion and clearing of large volume brain tissue and 60 - 100 µm sagittal brain sections we generally followed the proExM protocol published on expansionmicroscopy.org with minor modifications and recommendations for large tissue volumes.

Samples were first incubated in Acryloyl-X (AcX, Sigma-Aldrich, United States of America), which is dissolved in DMSO. Main purpose of the activation step is the introduction of acryloyl groups in the sample through AcX to allow for anchoring of the protein content to the polymer grid. Note, that the organic solvent DMSO is also used for delipidation in some clearing protocols, although in much higher concentrations (Bui et al., 2009). The duration of the activation step depends on thickness of the sections and was generally performed overnight (~12-15h for thin brain sections), with a minimum of 24h for thick (>500 µm) sections and whole HVCs.

Samples were next incubated in a monomer solution for 30 minutes to allow for equal diffusion of the solution prior to gelation, either in 48-well plates or small eppendorf tubes according to size. With as little delay as possible, incubated tissues or brain slices were transferred to a gelation chamber for conversion to a polymer gel. The recommended gelation chamber (Tillberg et al., 2016) works very well for sections and tissues up to 400 µm thickness by stacking up to three #1 coverslips for a total height of ~450 µm. For larger samples we trimmed 1 ml pipette tips to roughly 1.5 times the volume of the tissue and secured them on a coverslip wrapped in parafilm. The conical shape of the also chamber aids in recovering the hardened gel without damaging the embedded tissue. Gelation in Eppendorf tube caps as proposed by Cahoon et al. (2017) turned out impractical for extracting larger volumes of the hardened gel. As air impairs gelation, care should be taken to avoid entrapping of air bubbles when covering the chamber with parafilm.

After incubating the gels for 2h in a humidified chamber at 37º C, the hardened gels were trimmed to blocks containing the area or tissue of interest.

We generally measured expansion factors using a macroscope (Olympus, Japan). For samples exceeding the field of view of the macroscope expansion factors were measured by simply placing the gels on a millimeter grid.

For post-expansion staining we added DAPI in a concentration of 1:10000 during the last washing step of the expansion protocol for 15 minutes just before exchanging to the final imaging medium.

Expansion of large volumes of brain tissue and songbird tissue

The expansion protocol provides a unilateral expansion of tissues as originally demonstrated in sections and tissue volumes up to a thickness of 200 µm. Whether larger volumes of tissue with a higher degree of density inequality expand uniformly remains to be quantified. For example, thick sections of rat brain (>200µm) expand only partially using the standard proExM protocol supposedly due to the extremely dense corpus callosum. Here we show that sections and larger volumes of the songbird brain expand well despite its nuclear organization and heavy myelination. We did not observe any impartial expansions or any obvious inhomogeneity within the tissue at densely myelinated locations.

Imaging in the LaVision UltraMicroscope II

Expanded gels are generally fragile and transportation, movement or manipulation of the sample post gelation should be avoided or reduced to an absolute minimum. Expanded gel blocks were carefully transferred to the sample holder with a spatula and secured by placing

3% low melting agar at the corners or edges of the gel. Note that the fully expanded gels containing whole HVC exceeded the working range of the LaVision microscope stage
Imaging parameters used were single side illumination, 50-80 % laser power with 488 nm wavelength, smallest sheet thickness of 5 µm, sheet width ranging between 30 and 60, z-step size of 3-5 µm and dynamic focus.

Imaging in the Zeiss Z.1

We filled small 1 ml syringes with 3% agar and trimmed it in a way that created a flat surface that can be positioned perpendicular to the imaging path. The expanded sample was carefully positioned on the flat surface and secured in place with 1% agar (Figure 2g). Note that lower concentrations or only partially hardened agar can be insufficient to hold the sample in place.
Imaging parameters used were dual side illumination, 70-80 % laser power of the solid state diode lasers with wavelength of 405 nm and 488 nm, smallest sheet thickness of 5 µm and z-step size of 1-3 µm.
All datasets were produced with a Plan Apochromat 20x, 1.0 NA water dipping objective with a working distance of 2.4 mm.

Methodological and general considerations

(Pitfalls and recommendations)
With the larger volumetric size of the tissue to expand, handling becomes increasingly difficult with mounting the fully expanded gel for imaging presenting the greatest challenge. During the final water exchange steps we observed a partial collapse of the gel block due to gravitational pull when outside of water. Although the gel appears generally quite strong and no obvious fracturing occurs, we cannot exclude the possibility of shear forces introducing artifacts on the cross-linked content of the hydrogel. We therefore propose to execute the final expansion steps in larger volumes of deionized water with container diameters close to the expected final size, and only replacing parts of the water always leaving the gel fully submerged. Accordingly, movement of the final gel should be minimized. In imaging situation where the stage with the sample itself is moved, which is the case in nearly all light sheet setups, inertia of the gel might cause movement in larger gels. While small portions of gel have been successfully mounted on sample holders with minute amounts of super glue, we found glue to be impractical or impossible to immobilize large gels or for vertical mounting approaches as in the Z1. We find that placing small amounts of up to 3% low melting agarose around the edges of the gel to secure it in place is sufficient. Note that fast lateral movements during initial inspection of the sample should be avoided in order to minimize shear forces. Where possible, try to execute the final steps in the imaging container itself and immobilize the gel by replacing parts of the water with low concentration low melting agarose or phytagel (Sigma-Aldrich, United States of America), or cast another acrylamide gel around the expanded sample like suggested by Chen at al. (2016).

Although in some cases we observed wedging effects when mounting small gels by placing the agar right next to the gel, we did not observe this when placing the agar right onto the gel or in larger blocks of gel. It is possible however that the contact with the hot agar itself introduces some instant shrinking artifacts at the gel-agar interfaces. This is potentially not relevant if enough excess gel is around the tissue. However, if setup constraints prohibit excess embedding consider quantifying the extent of distortion by imaging linear structures close to the tissue surface pre and post expansion.

Supplementary movie S1.

Animation of an expanded HVC volume rendering.

Supplementary Figure S2.
Discernibility of Somata in the Ultra Microscope II dataset.

(**A**) Shows a digital section of the volume in Figure 1G. (**B**) Shows maximum intensity projection around the region highlighted in (A). Somata that appear very close (**C**) can be easily distinguished by rotation of the 3D dataset (**D**).
Scale bars (A) 250 µm, (B) 100 µm, (C & D) 50 µm.

Supplementary Figure S3.
Comparison of ExLSM to confocal microscopy.

(A) Shows a different rotation of the spiny dendrite in Figure 2C imaged with the 20x objective in the Zeiss Z.1. (**B**) Shows a maximum intensity projection of the same neuron type imaged in a Leica SP8 confocal microscope. The dataset in (B) has been deconvolved with Huygens while (A) is a direct visualization of the raw data.
Scale bars 5 µm.

References

Bui, A. K., McClure, R. A., Chang, J., Stoianovici, C., Hirshburg, J., Yeh, A. T., & Choi, B. (2009). Revisiting optical clearing with dimethyl sulfoxide (DMSO). *Lasers in Surgery and Medicine: The Official Journal of the American Society for Laser Medicine and Surgery*, *41*(2), 142-148.

Cahoon, C. K., Yu, Z., Wang, Y., Guo, F., Unruh, J. R., Slaughter, B. D., & Hawley, R. S. (2017). Superresolution expansion microscopy reveals the three-dimensional organization of the Drosophila synaptonemal complex. *Proceedings of the National Academy of Sciences*, *114*(33), E6857-E6866.

Chen, F., Wassie, A. T., Cote, A. J., Sinha, A., Alon, S., Asano, S. (2016). Nanoscale imaging of RNA with expansion microscopy. *Nature methods*, *13*(8), 679.

Tillberg, P. W., Chen, F., Piatkevich, K. D., Zhao, Y., Yu, C. C. J., English, B. P., ... & DeGennaro, E. M. (2016). Protein-retention expansion microscopy of cells and tissues labeled using standard fluorescent proteins and antibodies. *Nature biotechnology*, *34*(9), 987.
