# Supplementary figures and images for: Expansion Light Sheet Microscopy Resolves Subcellular Structures in Large Portions of the Songbird Brain
Source: Front Neuroanat. 2019 Jan 31;13:2. doi: 10.3389/fnana.2019.00002 (PMC6365838; doi:10.3389/fnana.2019.00002)

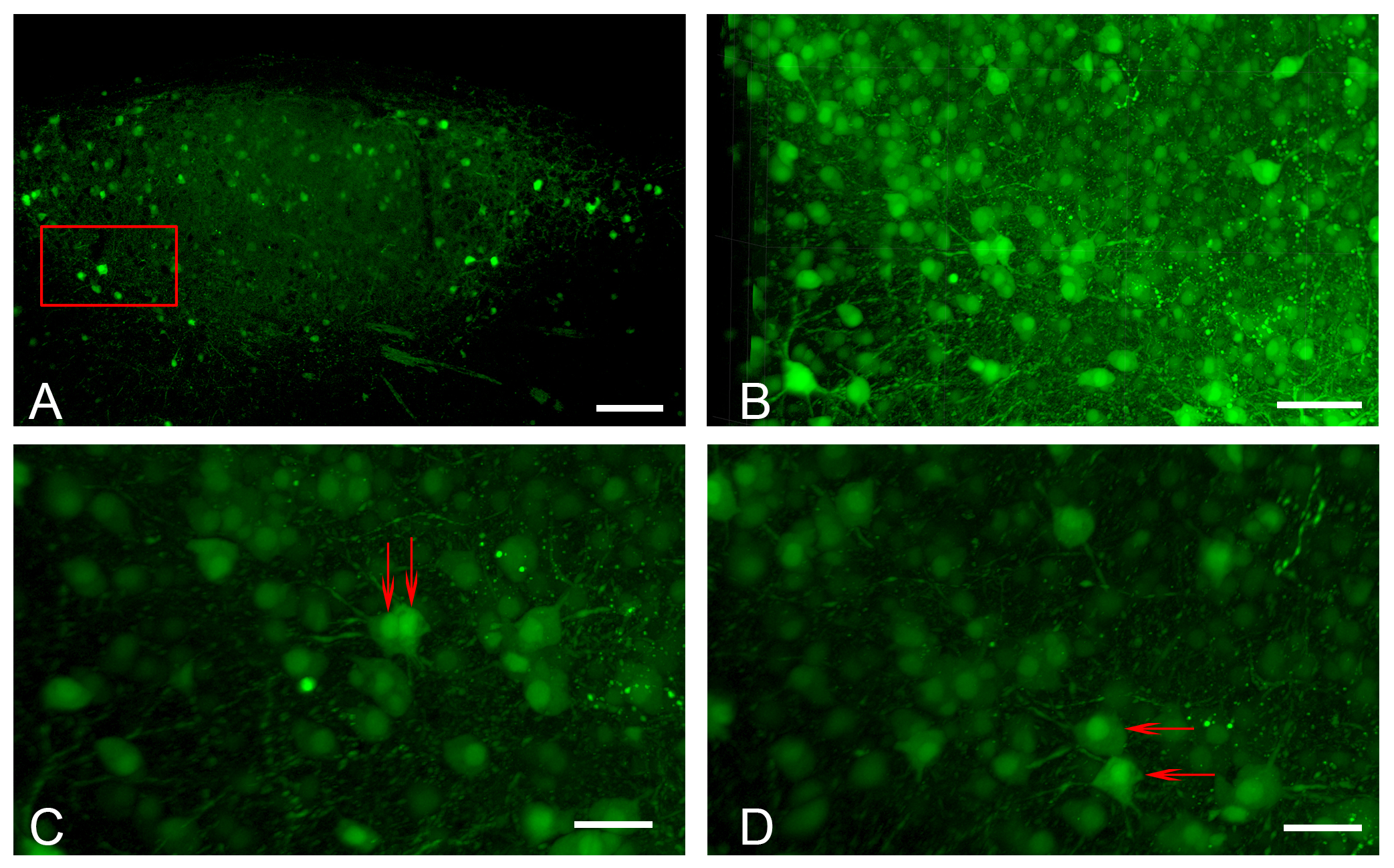

Supplement: Supplementary file 3 [file Image_1.JPEG]

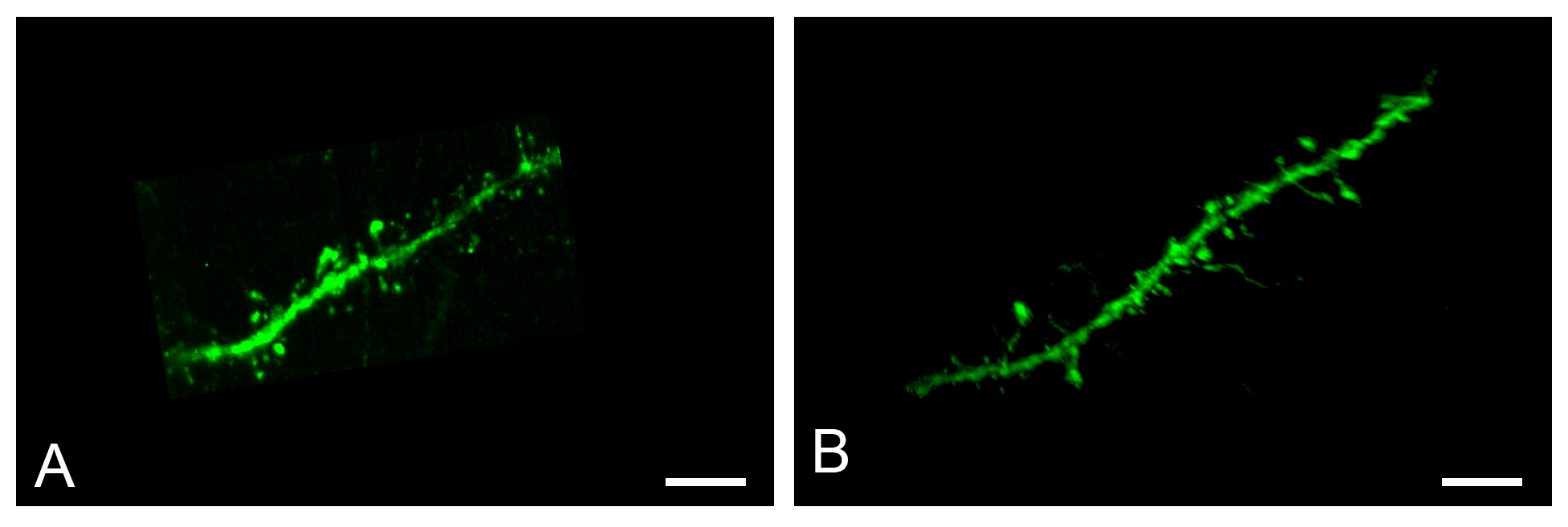

Supplement: Supplementary file 4 [file Image_2.JPEG]
